# Functionally graded titanium implants: Characteristic enhancement induced by combined severe plastic deformation

**DOI:** 10.1371/journal.pone.0221491

**Published:** 2019-08-23

**Authors:** Shokouh Attarilar, Mohamad Taghi Salehi, Khaled J. Al-Fadhalah, Faramarz Djavanroodi, Masoud Mozafari

**Affiliations:** 1 Material Science and Engineering Department, Iran University of Science and Technology, Tehran, Iran; 2 Department of Mechanical Engineering, College of Engineering & Petroleum, Kuwait University, Kuwait City, Kuwait; 3 Department of Mechanical Engineering, College of Engineering, Prince Mohammad Bin Fahd University, Al Khobar, Saudi Arabia; 4 Department of Mechanical Engineering, Imperial College London, London, United Kingdom; 5 Lunenfeld-Tanenbaum Research Institute, Mount Sinai Hospital, University of Toronto, Toronto, ON, Canada; University of Vigo, SPAIN

## Abstract

Commercially pure titanium was processed by equal channel angular pressing (ECAP) and surface mechanical attrition treatment (SMAT) for the purpose of developing functionally graded titanium used for implants and a gradient structure including nanostructured, deformed and undeformed zones were produced on the samples. In particular, it was aimed to design the gradient-structure in the titanium with enhanced properties by applying 4 ECAP passes to form bulk structure of ultrafine-grains and subsequently subjecting SMAT to the surface of ECAPed samples to produce nanostructured surface region. Microstructural examination was made by electron back scatter diffraction (EBSD). Also, microhardness, nanoindentation, topography, roughness and wettability were evaluated. To examine the biological response, human osteosarcoma cells were cultured in contact with the samples in various time periods and morphology change, cell viability and alkaline phosphate activity were conducted also cell morphology was monitored. EBSD showed development of ultrafine-grained structure after 4 passes of ECAP with an average grain size of 500 nm. Applying SMAT resulted in additional refinement in the ECAP samples, particularly in the subsurface regions to a depth of 112 μm. Furthermore, the SMATed samples showed an enhancement in roughness, wettability and hardness magnitudes. Viability enhanced up to 7% in SMATed _+_ ECAPed sample, although the acceptable cell adhesion, improved cell differentiation and mineralization were seen. The combined use of ECAP and SMAT has shown a good potential for optimizing the design of modern functionally graded medical devices and implants.

## 1. Introduction

Titanium has been considered as one of the most used biometals for dental roots and orthopedic prosthesis due to its favorable corrosion resistance, biocompatibility, and usage as a replacement for hard tissues [1]. One major disadvantage of pure titanium is the low mechanical properties, as compared to Ti-6Al-4V (Ti64 alloy) and other materials used in biomedical applications. The tensile strength and fatigue endurance limit of pure Ti are relatively low [2]. In this regard, alloying can significantly improve the mechanical properties of Ti, but in the case of Ti64 alloy, there are great concerns about the adverse effects of released aluminum and vanadium ions which lead to cytotoxicity and influence the cellular behavior such as osteoblast metabolism and differentiation [3] and even, DNA and nuclear damage [4]. Hence, alloying is not an ideal procedure for mechanical improvement of biomaterials.

Severe plastic deformation (SPD) methods have been successfully applied for improvement of mechanical properties by means of grain refinement to submicron level, to produce so called ultrafine-grained (UFG) structure. In particular, SPD methods can improve the strength and fatigue resistance of pure titanium to levels that exceed those reported for Ti64 [5], [6]. Recently, SPD methods have been applied for several types of biometals and alloys such as titanium [7], magnesium [8], stainless steel [9], AZ31 magnesium alloy [10], and NiTi shape memory alloy [11]. The main purpose of SPD methods is to intorduce high amount of plastic strains in the bulk of the material, via combined action of compressive and shear stresses, without any considerable dimensional changes. This typically requires an increase in the free energy of the polycrystalline material and thus generating much more crystal defects and grain boundaries. Consequently, it is possible to repeat the process and apply more strains to attain UFG structure, and in some cases nanostructured (NS) materials, with superior mechanical properties [12]. There are various techniques depending on the processes and shape of the samples. This includes equal channel angular pressing (ECAP) [13], planar twist extrusion (PTE) [14], and equal channel forward extrusion (ECFE) [15] for bulk samples. Accumulative roll-bonding (ARB) [16] and constrained groove pressing (CGP) [17] is used for the case of sheet samples and tubular channel angular pressing (TCAP) [18] for tubular samples. More recently, the fundamental of SPD processes have been introduced for grain refinement of the surface which called surface severe plastic deformations (SSPD). They involve several techniques which are mainly split from the shot peening method with higher impact kinetic energy; hence, they are also called severe shot peening (SSP) methods. Some of the most popular techniques are ultrasonic shot peening (USSP) [19], surface nanocrystallization and hardening (SNH) [20], high-energy shot peening (HESP) [21], and surface mechanical attrition treatment (SMAT) [22].

The aforementioned SPD methods have the potential to fulfill the requirements for the production of bulk biometallic products. The grain refinement via SPD, particularly ECAP, has been shown to enhance the mechanical strength and biocompatibility of titanium implants [6]. This also found to be applicable on other metals such as stainless steel [23], and AZ31 magnesium alloy [24]. Valiev et al. produced dental implants from the SPDed pure titanium with the trademark of Nanoimplant® [25]. Also, the increased osteoblast adhesion on ultrafine grained/nanophase SPDed Ti and Ti64 alloy in comparison with their coarse-grained (CG) counterparts was reported by Yao et al. [26]. They showed that the fabrication of nanostructured pure Ti by SPD not only produces a material with superior mechanical properties but also shows that it is a promising technique for producing miniature implants [27]. Additionally, the application of SPD on commercially pure titanium was shown to produce nano-structure capable of enhancing protein adsorption and assisting the proliferation and attachment of SaOS-2 cells [28]. The better biological response was related to physicochemical properties of the oxide layer, high density of non-equilibrium defects, increment of charged sites with the potential of molecular interaction.

Beside the bulk attributes of material, the enhanced surface properties and characteristics play a decisive role since most of the failures and cracks initiate from the exterior layers of samples such as wear, corrosion, and fatigue [29]. Also, the surface directly control the cell-substrate interaction; therefore, modifications of topography, roughness [30], wettability [31], and other surface parameters are of crucial significance. Bio-inspired NS materials (with surface structures less than 100 nm) can be essential to resolving current problems associated with the titanium-based implants. The enhanced cellular responses on NS Ti can lead to a stronger initial bio-integration with surrounded tissue to increase both the lifetime and bonding between tissues and implant surfaces. Interestingly, the so-called surface severe plastic deformation (SSPD) methods can optimally be used to meet surface qualifications for biometals. It has been shown that SSP treatment on AZ31 magnesium alloy produced the NS surface layer with increased roughness of 150%, microhardness about 133%, and wettability up to 20%. This was due to the formation of a surface layer with compressive residual stresses compared to the as-received CG sample. It was also, shown that no cytotoxicity was observed after day seven [24]. Better stem cell response, fatigue life, hemocompatibility, high surface hardness, and compressive residual stress in SMATed Ti was reported by Bahl et al. [32] which indicate that nanostructuring by SMAT technique is a promising procedure for implant design, especially for orthopedic and cardiovascular applications.

Yang et al. [33] developed a hierarchical structure in pure titanium via cryorolling and SMAT processing that resulted in the improvement of work-hardening ability without any strength sacrificing. A graded microstructure was observed between the successive layers which consists from three different layers namely an amorphous/nanocrystallite (A/NC) layer, an inner nanograined (NG) layer with the respective approximate thickness of 30, 60 μm, and a UFG core. The juxtaposition of multiple layers and the micromechanically graded structures caused a better stress redistribution which ultimately produced increased resistance to failures and improved mechanical properties. There are other investigations that confirm formation of the gradient structures by SMAT processing, including steel [34], Ti [35], Al [36], and Cu [37], [38]. It is reported that gradient structures of metals lead to an intrinsic synergetic strengthening effect which is much higher than the summation of separate gradient layers. This is probably due to macroscopic stress gradient and may be due to bi-axial stress generation by mechanical incompatibility between layers [39]. Although there are various studies about surface severe plastic deformation methods, investigations about the gradient structures and its effect on the mechanical, surface, electrochemical, and biological behavior are limited. Therefore, in the current study the ECAP and SMAT processes was combined for the first time and as a new scheme for systematic and simultaneous design of both the surface and the bulk of material for potential use in commercially pure Ti implants. The produced samples have micromechanically graded structure, possessing superior mechanical properties. The microstructure and mechanical properties were studied through the thickness of material due to its hierarchical structure. Also, the surface parameters and biological response of the samples was investigated. This new procedure because of its improved surface parameters in combination with high mechanical properties elevated the osseo-integration of cells with the substrate, which is from crucial importance due to ever increasing need for durable implant materials which reduces the need to replace implants and secondary surgeries.

## 2. Material and methods

### 2.1. Processing by severe plastic deformation

**Fig 1:**
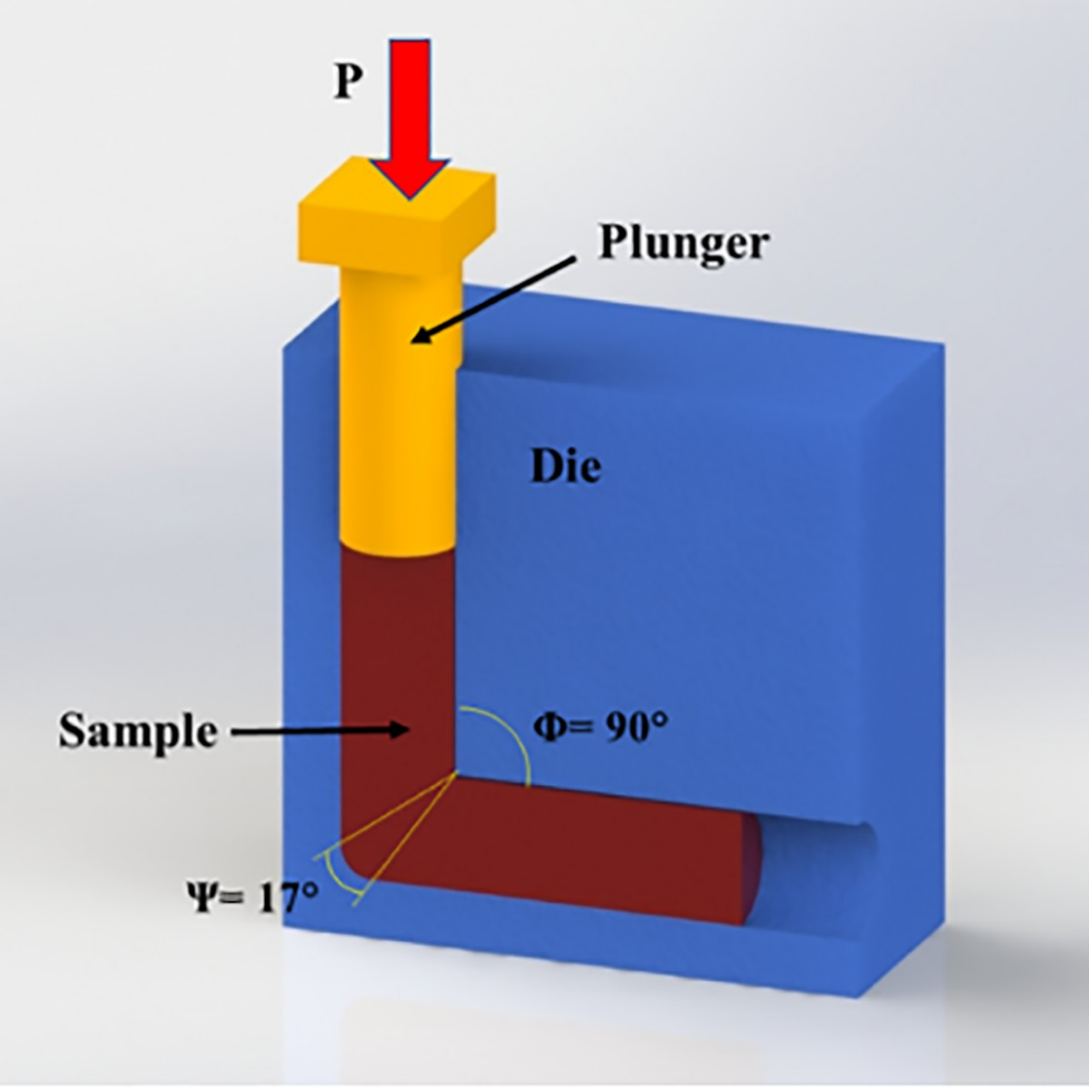
Schematic illustration of ECAP die including channel angle Φ and corner angle Ψ.

**Fig 2:**
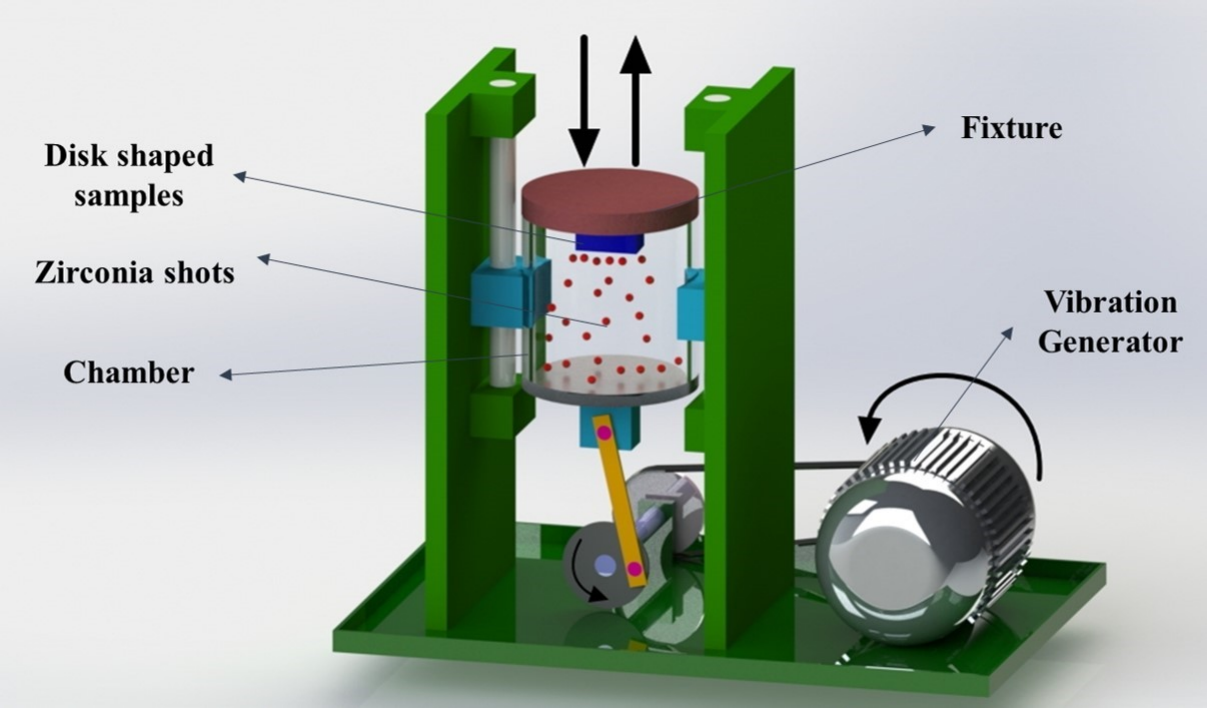
A schematic of surface mechanical attrition treatment (SMAT) device, indicating different parts of device.

Commercial pure titanium (CP Ti Grade 2) bars with 99.5% purity was provided by Baoji Qicheng Non-ferrous Metals Co., Ltd. and sectioned to 70 mm length and 20 mm diameter specimens and annealed at 800°C for 1 hour. Chemical composition of the used CP Ti was (wt%) 0.3% Fe, 0.25% O, 0.1% C, 0.03% N, 0.015% H and Ti (balance). Subsequently, the samples underwent ECAP processing conducted at 450°C up to 4 passes. The custom-built ECAP die as shown in Fig 1 consists of two equal channels with 20 mm diameter, channel angle of 90°, outer corner angle of 17°, and 2 mm/s ram speed by route B_C_ in which the sample is rotated 90° clockwise around its axis. Route B_C_ has been considered as the most effective one for microstructural refinement using ECAP processing [40]. A molybdenum disulfide-based solid (MoS_2_) lubricant was provided from Luoyang Exploiter Molybdenum Co., Ltd, and used to reduce the effects of friction from die walls. The initial (annealed) and ECAPed samples were sectioned using electric discharge machining (Mitsubishi electric MP1200) to disks of 20 mm in diameter and 2 mm in thickness for further processing by SMAT. The disks were later grinded with SiC abrasive papers up to 600 grit to ensure surface uniformity. Fig 2 is a schematic of the custom-built SMAT device, which consists of a stainless-steel chamber of 90 mm height and 80 mm diameter that moves in a reciprocating motion as driven by an electrical motor. Through this type of movement, hard beads (shots) can directly impact one side of the surface in a random manner. A detailed description of the device can be found elsewhere [41]. A disk fixture with a capacity to hold two SMAT disks was designed and manufactured. Ceramic zirconia beads from ZirPro’s Zirblast® with 5 mm diameter, 700 Hv, 3.85 g/cm^3^ specific gravity, and composition of 60–70% ZrO_2_, 28–33% SiO_2_ and Al_2_O_3_ <10% were used in order to prevent the entry of toxic elements to the sample surface which is very usual in common steel beads. These beads have chemically inactive nature, white color, and very smooth surface and a hundred of these ceramic zirconia shots were placed in the chamber. The time taken to produce SMAT disks were 2 hours. To prevent overheating of the disks, the machine was turned off for 10 minutes every half an hour. The shots impact angle was at 90° and frequency was fixed to 6 Hz which result in the approximate shot speed of 3.6 m/s. There were four different samples in this study; annealed (A), SMATed annealed (SA), 4 passes ECAPed (4E) and SMATed four passes ECAPed (S4E) sample which are listed in Table 1.

**Table 1:** The condition and name of different CP Ti samples.

Sample name	condition	Used beads and SMAT duration	ECAP pass numbers
A	Annealed Ti	-	0
SA	SMAT processed annealed Ti	Zirconia– 2 hours	0
4E	Four passes ECAPed Ti	-	4 passes
S4E	SMAT processes four passes ECAPed Ti	Zirconia– 2 hours	4 passes

### 2.2. Mechanical properties

Microhardness measurements were performed on grinded and polished samples in annealed and ECAP conditions using ShabSari M5 microhardness tester. Also, a coupon of SMAT disk in annealed condition was cut from the middle and polished. The microhardness indentations were made through the thickness of the samples in an increment of about 75 μm in A and SA samples. All Vickers microhardness measurements were done with 500 gf load and dwell time of 15 s. It was reported that nanoindentation method has a capability to determine the mechanical properties of materials with nanostructure surface [42]. Also, this method can be precisely detect the transition between nanostructure surface and the coarse grained matrix [43] hence the nanoindentation measurement was performed. A nanoindentation tester (model: Hysitron-Ti700 UBI) was used. Prior to testing, nanoindentation calibration was conducted following the Pharr-Oliver calibration method and using fused-silica standard sample [44]. Three-sided pyramid diamond Berkovich indenter was used to make the indentations, with radius of curvature of 150 nm and inclined angle of 142.3°. The nanoindentation measurements were made using 20 mN force (to minimize any size indentation effect) and a line profile with an indentation increment of 5 micrometers, starting from the SMAT surface towards the subsurface layers (up to 230 micrometers). Five indentation lines were used for each sample and the average results of hardness and reduced modulus were reported for SMAT processed 4 passes ECAPed (S4E) sample.

### 2.3. Surface and microstructural examination

Topography and roughness of specimens were examined by atomic force microscopy (AFM), model ARA-AFM 0101/A, using non-contact condition on an area of 20 × 20 μm^2^. Also, wettability and contact angle measurements were performed by Sessile-drop method at room temperature and distilled water was used as the wetting liquid. Drop density was 1 g/cm^3^ and the diameter of the needle was 0.793 mm. Contact angles of the air-water-substrate interface was measured three times via a digital camera and image analysis software, finally the average contact angles were reported. In order to study the surface condition after SMAT process, the samples were examined by scanning electron microscopy (SEM), model TESCAN VEGA. For this purpose, SA sample were cut in the middle and in order to analyze the depth of material, its cross-section was studied by SEM with voltage of 20 kV. In addition, the microstructure of SA and 4E samples was studied by electron backscatter scanning diffraction (EBSD). For EBSD characterization, coupons were cut from the disks to examine the microstructure at the cross-section. The coupons were prepared by coarse and fine grinding with SiC sandpapers up to 5000 grit, following by chemo-mechanical polishing with colloidal silica suspension and hydrogen peroxide. As a final polishing step, the EBSD coupons were prepared using an electro-polishing unit (model: Struers-LectroPol5) with Struers A3 electrolyte. The conditions for electro polishing include voltage of 35 V and electrolyte temperature of about 5°C. The EBSD detector and post-processing software (model: Oxford-Aztec) were attached to SEM (model: JEOL 7001F-JSM). The EBSD maps using an operating voltage of 20 kV and two magnification and step sizes were used (i) low-magnification (X500) observation using a step size of 500 nm and (ii) high-magnification (X 2000) observation using a step size of 60 nm. The misorientation angle distribution statistics was analyzed, employing a critical misorientation angle of 15° to differentiate low-angle boundaries (LAGBs) from high-angle boundaries (HAGBs). The grain boundaries were presented in EBSD maps such that LAGBs are depicted as grey lines and HAGBs as black lines. Grain size distribution and average grain size was determined using EBSD post-processing Aztec software.

### 2.4. Cell viability, alkaline phosphatase (ALP) activity, and cell attachment

G292 cells, a homo sapiens human osteosarcoma cell line provided from Pasteur Institute with adhesive nature were cultured on the surface of the sample after sterilization and cleaning by sequential ultrasonication in acetone and ethanol under the standard protocol of MTT tests. The culture media was Dulbecco’s Modified Eagle’s Medium (DMEM) from Sigma-Aldrich. The cell line was cultured in 100% humidity and 5% CO_2_, at 37°C conditions. The medium was changed every day and cells were passage through 0.05% trypsin/0.02% EDTA. For viability, cells were cultured for 1, 3, and 8 days and 1 day for the adhesion test. The conventional 96-well culture plates (n = 3 for each set) was used. The cells densities on the samples and control were analyzed via a UV spectrometer by a viable color change in the cells. The color absorbance was measured at 490 nm wavelength using microplate reader ELx808 Bio-Tek. In addition, cells were cultured on the samples for 1, 3 and 5 days and ALP activity was measured in 2-amino-2-methyl-1-propanol buffer, pH 10.3, at 37°C with p-nitrophenyl phosphate as the substrate. Enzyme activity was read at 405 nm by a microplate reader. The ALP activity was reported in terms of micromoles per minute per milligram protein. In order to assay the morphological characteristics, cells were grown on the samples for 1 day and subsequently were washed with phosphate-buffered saline (PBS) and then fixed by 2.5% glutaraldehyde and dehydrated in ethanol-water baths graded series to 100% and finally they were studied through the SEM device.

## 3. Results and discussion

### 3.1. Microstructure

**Fig 3:**
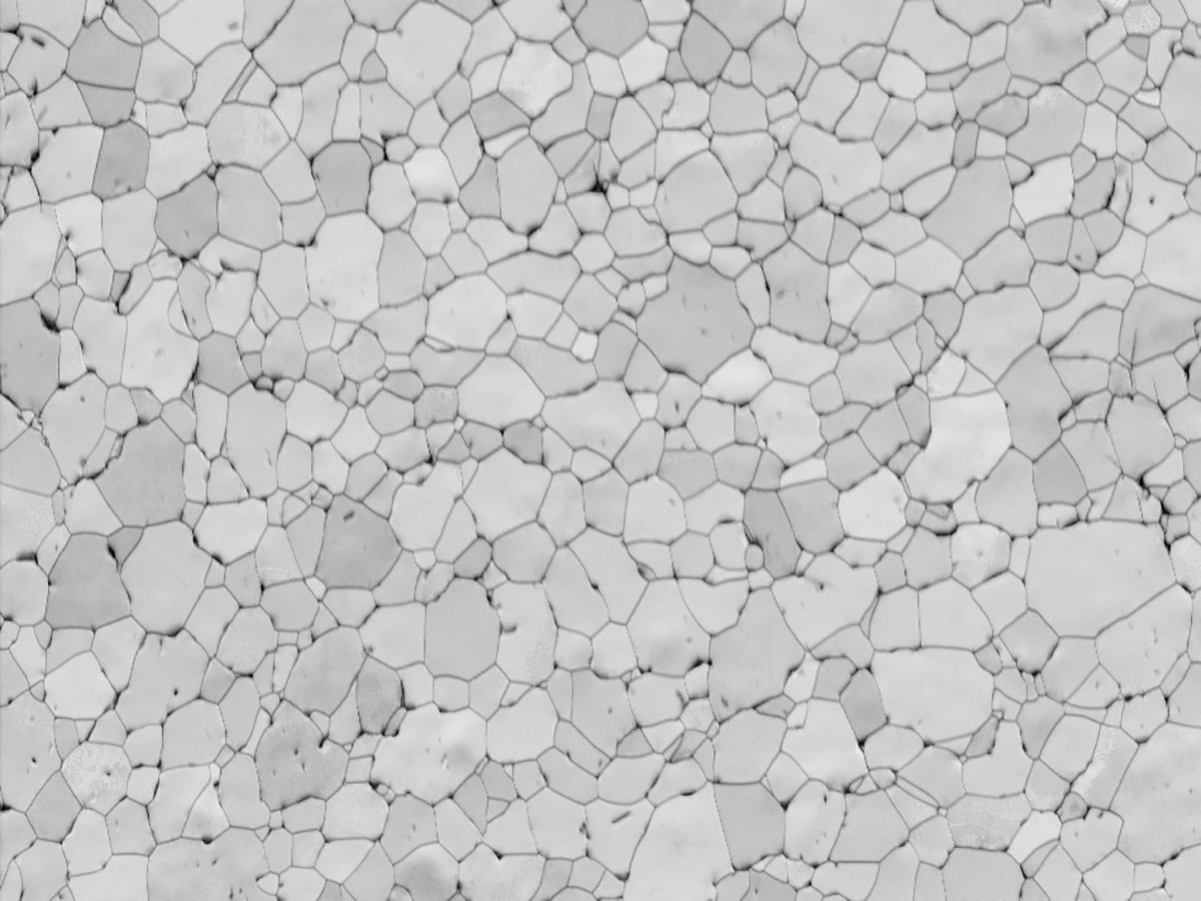
Band contrast of the CP Ti sample in the annealed condition with average grain size of 24 μm.

Fig 3 shows the microstructure of annealed CP Ti consisting of α-grain, with an average grain size of ~24 μm.

**Fig 4:**
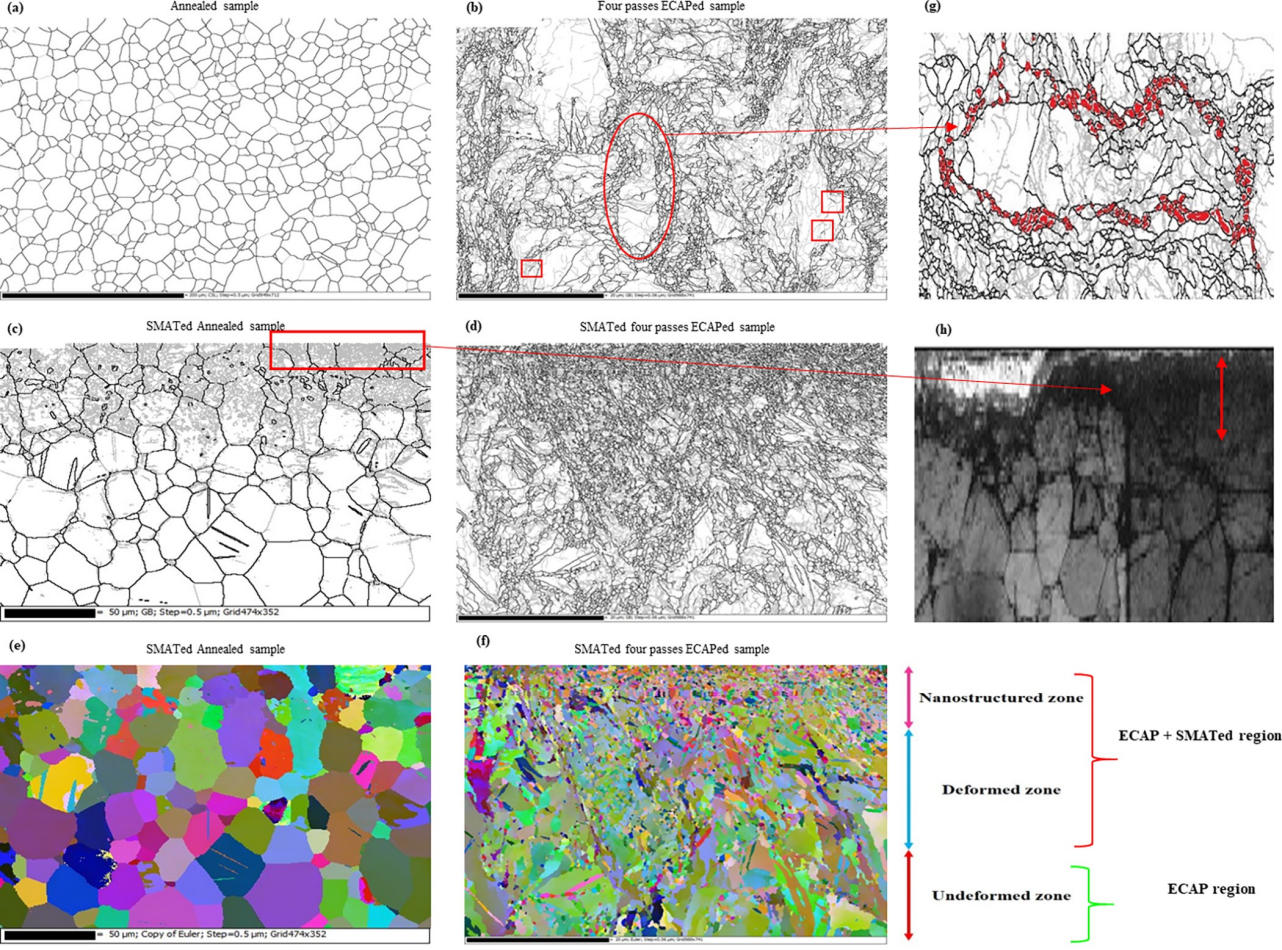
The EBSD grain boundary and Euler maps of Ti samples showing the different structural regions in the depth of samples produces by SMAT and ECAP processing, these regions are entitled from top to bottom as nanostructured, deformed and undeformed zones. The undeformed zone is not affected by SMAT. Black lines showing HAGBs and gray lines are LAGBs; (a) sample A; (b) sample 4E grain boundary maps before SMAT processing; (c) sample SA; (d) sample S4E grain boundary maps showing the effect of SMAT processing; (e) and (f) corresponding Euler maps of (c) and (d); (g) the necklace structure formation in 4E sample, magnified and rotated from the illustrated red oval region of 4E sample in (b), the squared regions shows the formation of incomplete HAGB segments; (h) band contrast map of magnified rectangular region in (c) showing the unindexed nanograined region and the red arrow shows the thickness of unindexed region.

Fig 4(A) and 4(B) shows EBSD grain boundary maps of Ti samples in the annealed and ECAP conditions. The annealed sample contains coarse equiaxed grains of α-phase with average grain size of 24 μm. The microstructure is generally free from low angle grain boundaries (LAGBs) with misorientation angles less than 15° indicating that the sample was fully annealed. Upon applying 4 passes of ECAP, subgrain structure of LAGBs with gray lines strongly prevailed in the microstructure, Fig 4(B). Formation of high angle grain boundaries (HAGBs) with misorientation angles higher than 15° is shown to occur within the initial structure. It can be seen that some of these fine equiaxed grains with average grain size of ~250 to 400 nm is nucleated around the grain boundaries of elongated grains or at old grain boundaries which generally known as necklace structure [45], shown in Fig 4(G). The existence of necklace structure with very fine grains that are almost free of LAGBs gives some hints about grain refinement mechanism. It was confirmed that continuous dynamic recrystallization (cDRX) may lead to formation of necklace structures since the rapid occurrence of strain gradients near grain boundaries which causes large misorientations in proximity of the boundaries [45]. It was also confirmed that cDRX play an important role in grain fragmentation during ECAP process of CP Ti [46]. Dynamic recrystallization arises by ongoing subgrain rotations specifically in materials with insufficient number of slip systems like Ti. These subgarin rotations usually happens in juxtaposition of HGABs and proliferation of deformation leads to strain gradient generation from middle to the boundary of old grains hence some new grains with HAGBs are formed which results the necklace structure formation with equiaxed fine grains [45–49]. Another hint for cDRX activation is the existence of incomplete HAGB segments, shown with squares in Fig 4(B). It was known that these incomplete HAGB segments are gradually evolves from the LAGBs by continuous accumulation of dislocations which increases the misorientation, this phenomenon confirms the cDRX occurrence [50]. After four passes of ECAP, the average grain size due to cDRX reduces to ~500 nm, Fig 4(B). It can be claimed that grain fragmentation basically happens through cDRX mechanism but it does not reach its homogenous structure with fully fragmented condition because of low HAGBs fraction [51] so increased pass numbers would be beneficial.

In addition, Fig 4(C) and 4(D) presents EBSD maps of annealed sample and 4-passes-ECAP sample after applying SMAT. The microstructure is shown to contain different microstructural regions. The application of SMAT on annealed sample resulted in severe plastic deformation of the subsurface region to a depth of about 100 μm due to residual compressive stresses applied by SMAT, Fig 4(C). Grain refinement in this region was attained through dislocation cell formation by the gradual alteration of dislocation cell walls to HAGBs that can be achieved by progressing straining [52]. This is demonstrated by the large fraction of LAGBs in the subsurface region which are the direct result of elastoplastic strain gradients. These strain gradients lead to the development of a compressive stress field in the upper layers with specific thickness [53]. Formation of small grains of HAGBs and some twins is also found to occur, Fig 4(C). The average grain size of SA sample in the deformed region shown in Fig 4(C) does not considerably change due to SMAT and the population of HAGBs are almost same in this region. This is due to fact that grains must reach the critical shear value in order to fragment [54]. The variations in color of grains in the regions beneath the SMATed surface, shown in Fig 4(E), represents the difference in internal misorientations caused by SMAT [55]. There is also an evidence of deformation twin’s in regions below 100 μm, in which LAGBs were rarely formed. It was reported by Zhu et al. [35] that grain fragmentation through SMAT in α-Ti samples happens in five steps, firstly twins systems are formed and intersected, then due to high density of dislocations disoriented lamellae forms. The last steps include the subdivision of microbands into blocks and formation of polygonal grains then these polygonal grains fragmented to nanograins. Twin formation is observable in Fig 4(C) but due to unindexed regions in the very edge of top SMATed layer containing nanograins Fig 4(H), it is not possible to claim the occurrence of these five-step mechanism in SA sample. In the case of S4E sample, it is clear that further grain refinement occurred in the subsurface region below the SMATed surface (Fig 4(D) and 4(F)). This resulted in formation of fine nanostructured region with grain size below 250 nm. Beneath the nanostructured region, there is a deformed region with UFG structures with a high LAGBs fraction. With increasing distance from surface, the microstructure is shown to be lightly affected by SMAT. The grain fragmentation by SMAT mainly occurred in the upper subsurface regions and the grains are considerably smaller than the other zones also the grains in the SMAT effected regions have random distribution in both SA and S4E samples in Fig 4(E) and 4(F), this phenomenon is also reported by Bagherifard et al. [24]. It should be noted that subsurface regions below SMATed surface was shown to contain an unindexed region during EBSD measurements due to possible formation of nanostructured or amorphous region via SMAT processing Fig 4(H). Grain sizes in the order of the interaction volume size or smaller than band slope electron interaction cause un-indexing or dark regions [56]. It was reported that overlapping nano grains and multiple patterns in grain boundary area could lead to unindexed regions in EBSD [57].

**Fig 5:**
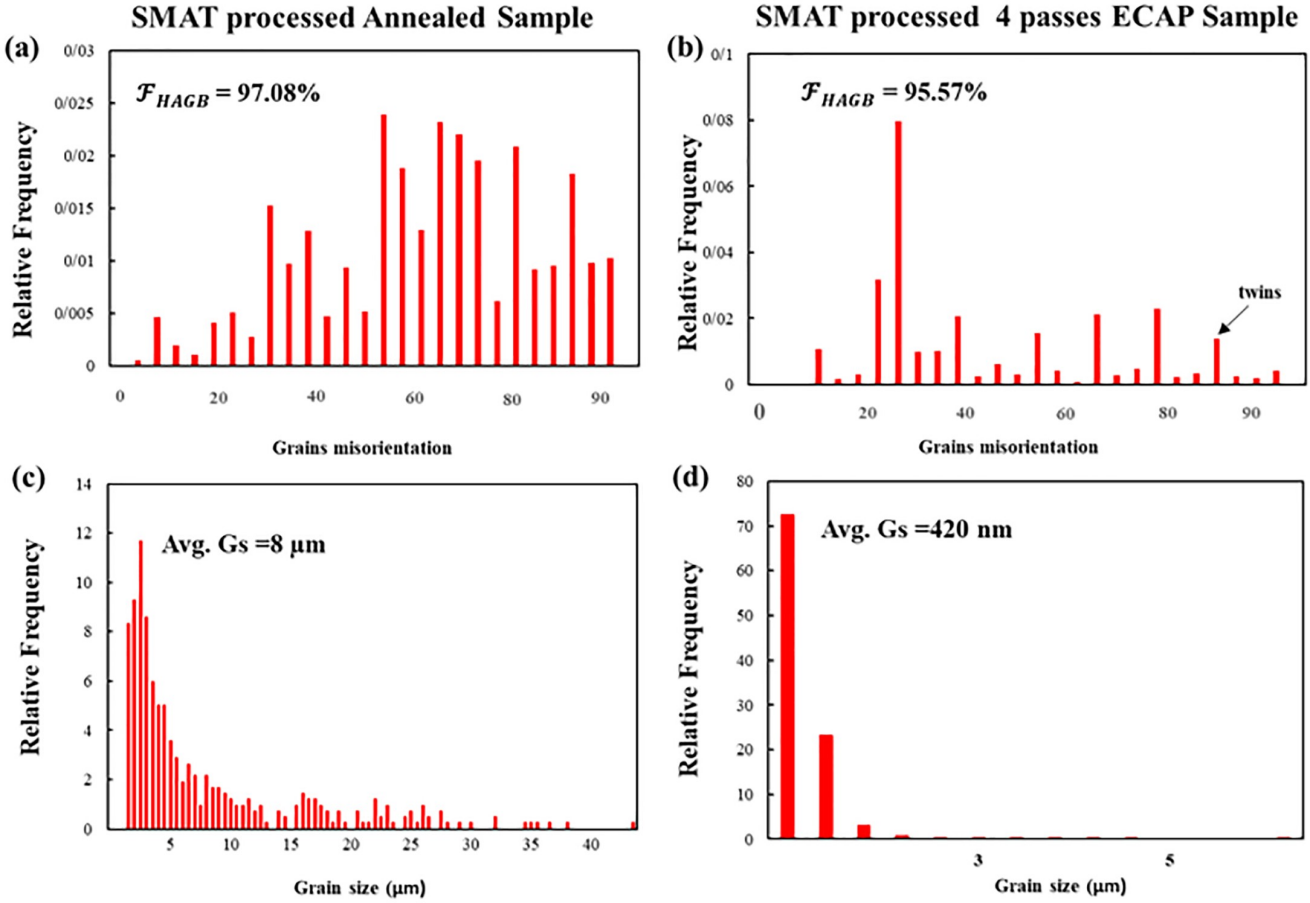
Misorientation and grain size distribution of SMATed samples; (a) and (b) misorientation; (c) and (d) grain size distribution of SA and S4E samples respectively in accordance to relative frequency. The black arrow shows the existence of twin boundaries.

Fig 5 shows the misorientation and grain size distribution of SMATed samples calculated by the grain area determination method [58], the values are also listed in Table 2. There is an abrupt shift of peaks to the left side in the S4E sample compared to sample A, Fig 5(C) and 5(D) showing the great refinement of the structure by reduction of average grain size from 8 μm to 420 nm; about 95% reduction which is induced through both ECAP processing and SMAT. The effect of SMAT on grain refinement is more severe in the ECAPed condition which is apparent from observation of Figs 4(C), 4(D), 5(C) and 5(D). Grain size distribution shows that there is not any trace of initial grains with an average grain size of 8 μm, this means that the microstructure is fragmented. In S4E sample most of the grains have sizes in the range of 400 nm and the HAGBs are dominant. There is a peak in the misorientation distribution of this sample around ~85° (marked by black arrow) that is an indicative of $\left\{10\bar{1\;}2\right\}$ tensile twin boundaries, this phenomenon was also reported by Qarni et al. [55]. The existence of these twins is representative of combined effect of deformation by both dynamic recrystallization and twinning mechanism. The $\left\{10\bar{1\;}2\right\}$ tensile twins are very usual since they need the least amount of twining shear and they can be activated readily [59]. Careful observation of Table 2 shows that the fraction of HAGBs are very low in 4E sample showing the unstable condition of this sample. SMAT processing significantly improves the amount of HAGBs confirming that SMAT causes a lot of defects, shear gradients and dislocations which increases the misorientation angles and finally transforms the LAGBs to HAGBs, this shows the beneficial effect of SMAT process on ECAPed Ti sample. The grain refinement through the combined application of ECAP and SMAT is favorable for the biological response of materials, since the grain refinement can provoke various bone type cells and leads to better proliferation and adhesion [27]. The standard deviation data also confirms the beneficial effect of SMAT processing that leads to production of the more homogenous structures in top layers of samples.

**Table 2:** The average grain size, fraction of HAGBs and standard deviation of different Ti samples calculated by EBSD data.

Sample Name	Average grain size	Fraction of HAGBs	Standard deviation
A	24 μm	~100%	9.43
SA	8 μm	97.08%	7.81
4E	500 nm	32%	0.42
S4E	420 nm	95.57%	0.29

### 3.2 Surface characteristics

**Fig 6:**
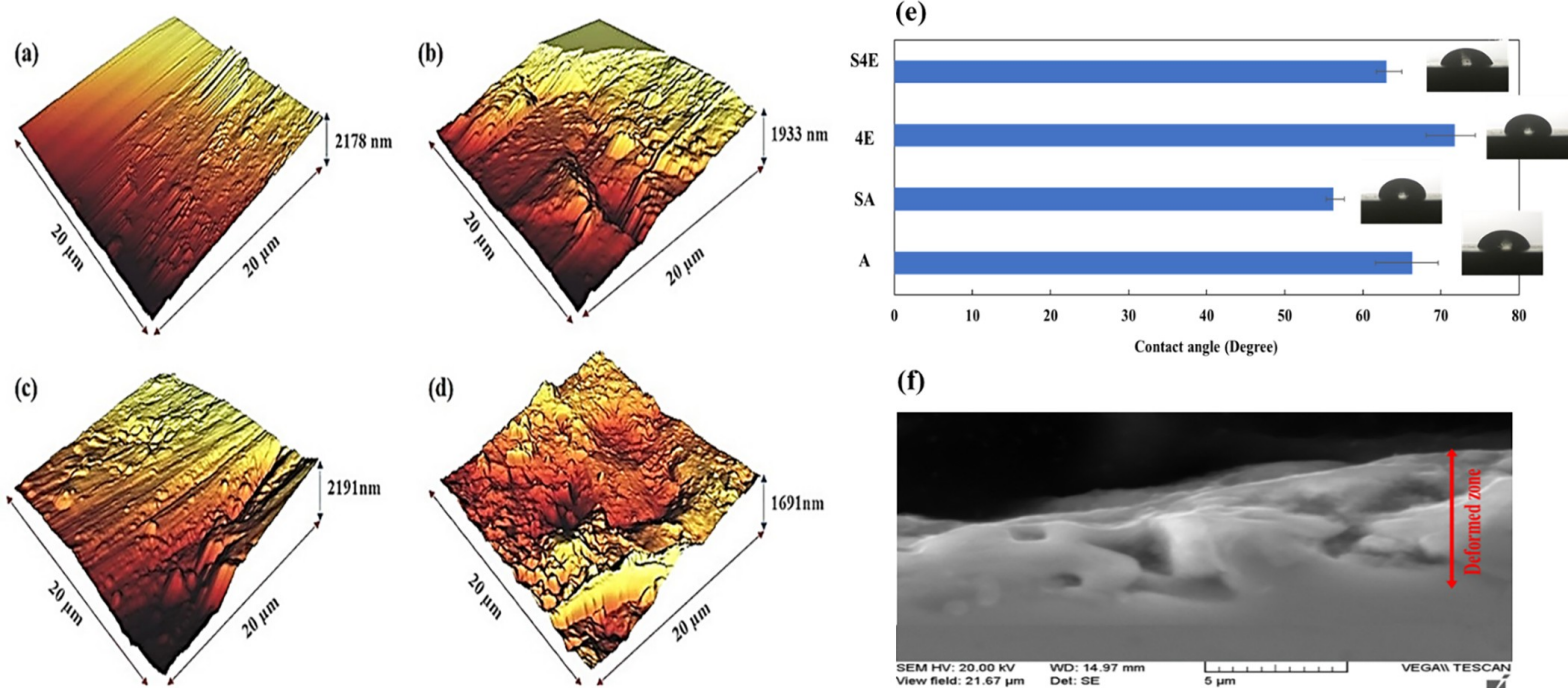
Surface properties of Ti samples; AFM topography of samples in 20*20 μm^2^ area, (a) A, (b) SA, (c) 4E, and (d) S4E sample; (e) contact angle results of Ti samples; (f) SEM micrograph of SMATed as-received Ti (SA) sample showing the SMAT effected zone full of micrometer ranged cracks and pores.

Fig 6 shows the various surface properties of different Ti samples. Fig 6(A)–6(D) represents the AFM topography of as-received and ECAPed samples in SMATed and non-SMATed conditions (SA, A, S4E and 4E samples). In this regard, Table 3 lists the roughness values in 20×20 μm^2^ area. As seen, the application of SMAT process induces 55.7% improvement in Rz value in SA sample and for the case of ECAPed sample, this magnitude is 88.6%. It is found that Rz can be a good candidate for estimating the existence of high peaks and valleys than the other parameters such as Ra which is the arithmetic average height parameter and Rq which measures the standard deviation of surface heights distribution [60]. These improvements in Rz values show the ability of SMAT process to induce a lot of peaks and valleys which can be the preferred sites for cell adhesion and provide regions for favorable mechanical bonding of implants with surrounding tissues. Also, it has the potential to enhance the bioactivity and early adsorption of the extracellular matrix (ECM) proteins [61]. It was reported that only surfaces with roughness values of less than 1 μm can have good cell proliferation regardless of wettability magnitudes [62]. Consequently, considering the values of Table 3, it can be claimed that SMAT processing is a good procedure to enhance the cellular behavior of Ti samples. Fig 6(E) shows the contact angles of different titanium samples, the values are also listed in Table 3. It is obvious that SMAT processing leads to lower contact angles which cause better wettability. In the annealed sample due to SMAT, contact angle is reduced by 16.5% and for the ECAPed condition, this value is 13%. It was confirmed that better wettability improves the interaction between implant substrate and the surrounded tissue [63]; hence, SMAT has also beneficial effects from the wettability aspect. Fig 6(F) represents the SEM image of SA sample with micrometer ranged cracks and pores which produces potential preferred regions for cell proliferation and adhesion. It is assumed that these pores and cracks enclose the cells and make a suitable place for cell adhesion and growth, especially in cells with adhesive nature-like osteoblasts [64]. It is believed that micro- and nano-structured surface pores and cracks can increase the specific surface area which leads to enhancement of surface reactivity with the surrounding ions, amino acids, and proteins, that finally determines the initial adsorption of calcium and phosphate ions as well as cellular behavior at the cell-implant interface [65].

**Table 3:** Contact angle and roughness and values of different titanium samples in 20×20 μm^2^ area.

Sample	Contact Angle	Ra (nm)	Rz (nm)	Rq (nm)
**A**	66.37°	9.907	78.45	71.44
**4E**	71.78°	10.53	117.4	75.95
**SA**	56.23°	10.51	139.1	75.77
**S4E**	63.06°	18.89	304.3	136.9

### 3.3. Through-thickness hardness development

**Fig 7:**
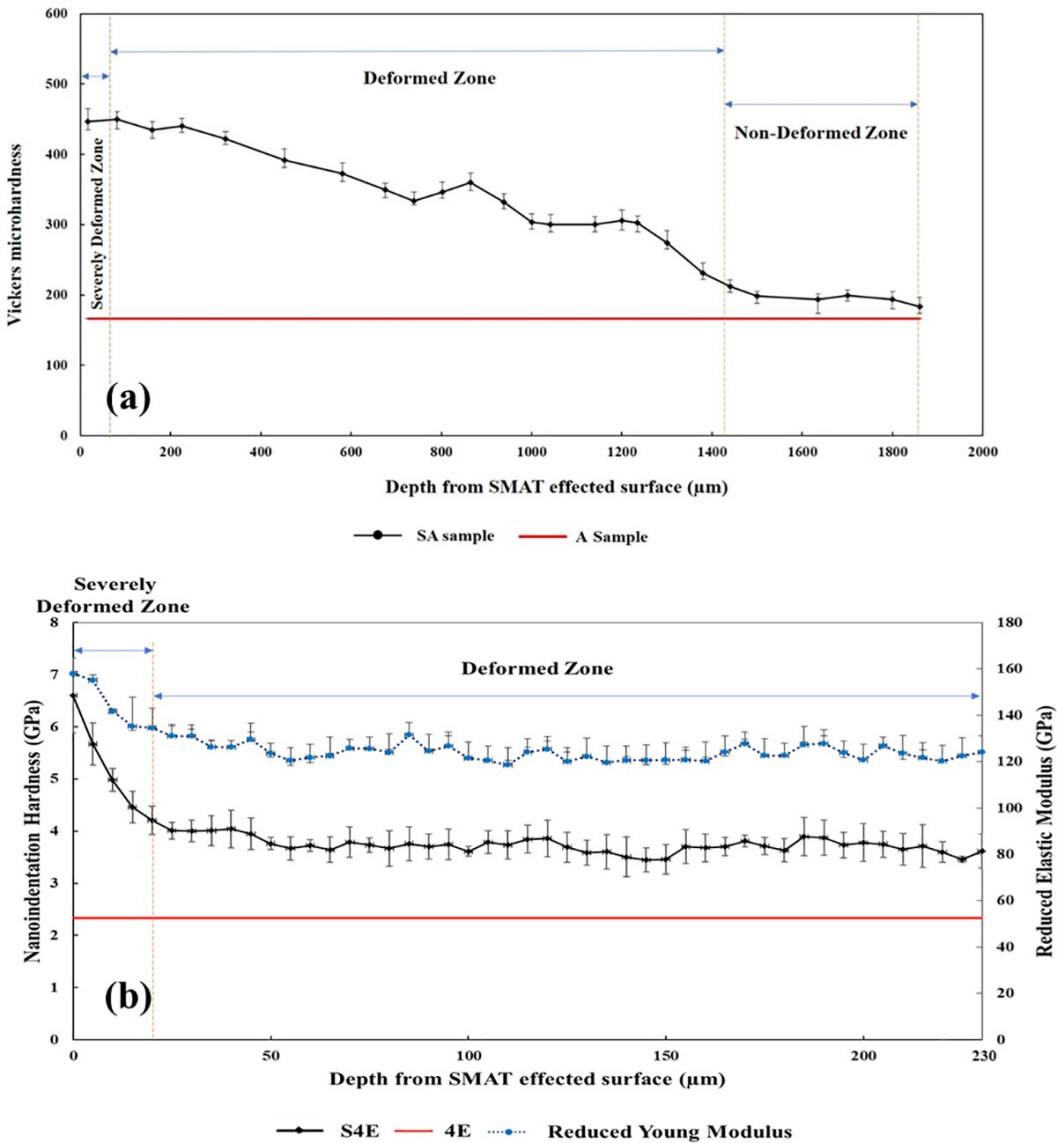
(a) Vickers microhardness values of the as-received sample with and without SMAT processing SA and A samples in different depths from the SMAT affected surface; (b) Nanoindentation hardness and reduced Young modulus (Er) results in depth from SMAT affected surface 4 passes ECAPed samples with and without SMAT processing S4E and 4E samples.

The through-thickness microhardness profiles for A and SA samples are shown in Fig 7(A), to a depth of 1800 μm of the SMATed surface. It can be observed that approximately in the 82 μm distance from the surface, there is a severely deformed zone which its Vickers microhardness value is 449, about 92% higher than the A sample with just 166 Hv. Also, there is a deformed zone from 82 up to 1440 μm from the surface with an average microhardness of 341 Hv, about 69% higher than the initial sample, and finally in distances more than 1440 μm, there is a non-deformed zone which its hardness is almost same as the initial sample with slight increment.

Fig 7(B) represents the through-thickness profiles of nanoindentation hardness and reduced Young modulus (Er) for S4E and 4E samples, plotted to a depth of 230 μm from SMAT effected surface. In the S4E sample, the severely deformed area approximately has 20 μm thickness and its average hardness is 5.18 GPa (528 Hv) about 75.6% more than 4E sample and also the average hardness of the deformed zone up to 230 μm is about 3.72 GPa (379.3 Hv) with 45% increment compared to 4E sample. In addition, the reduced modulus of 4E sample has a similar trend and it decreases as a function of depth from SMAT treated surface. The maximum value in the topmost surface is 158 GPa about 27% enhancement and then gradually decreases. It is claimed that microstructural gradation is the main factor responsible for these nanomechanical variations as reported in [66, 67]. It was reported that nanocrystalline materials may exhibit improved Er values [68]. Similarly, the hardness improvement in the topmost surface of SA and S4E samples can be related to microstructural refinement via SMAT in accordance with well-known Hall-Petch relationship [69, 70].

It should be mentioned that the thickness of severely deformed zone in S4E sample is lower than the SA one which is probably due to low efficiency of SMAT process in work-hardening of UFG substrates, since UFG materials have lower efficiency for dislocation accumulation [71].

### 3.4. Cell viability, alkaline phosphatase (ALP) activity, and cell attachment

**Fig 8:**
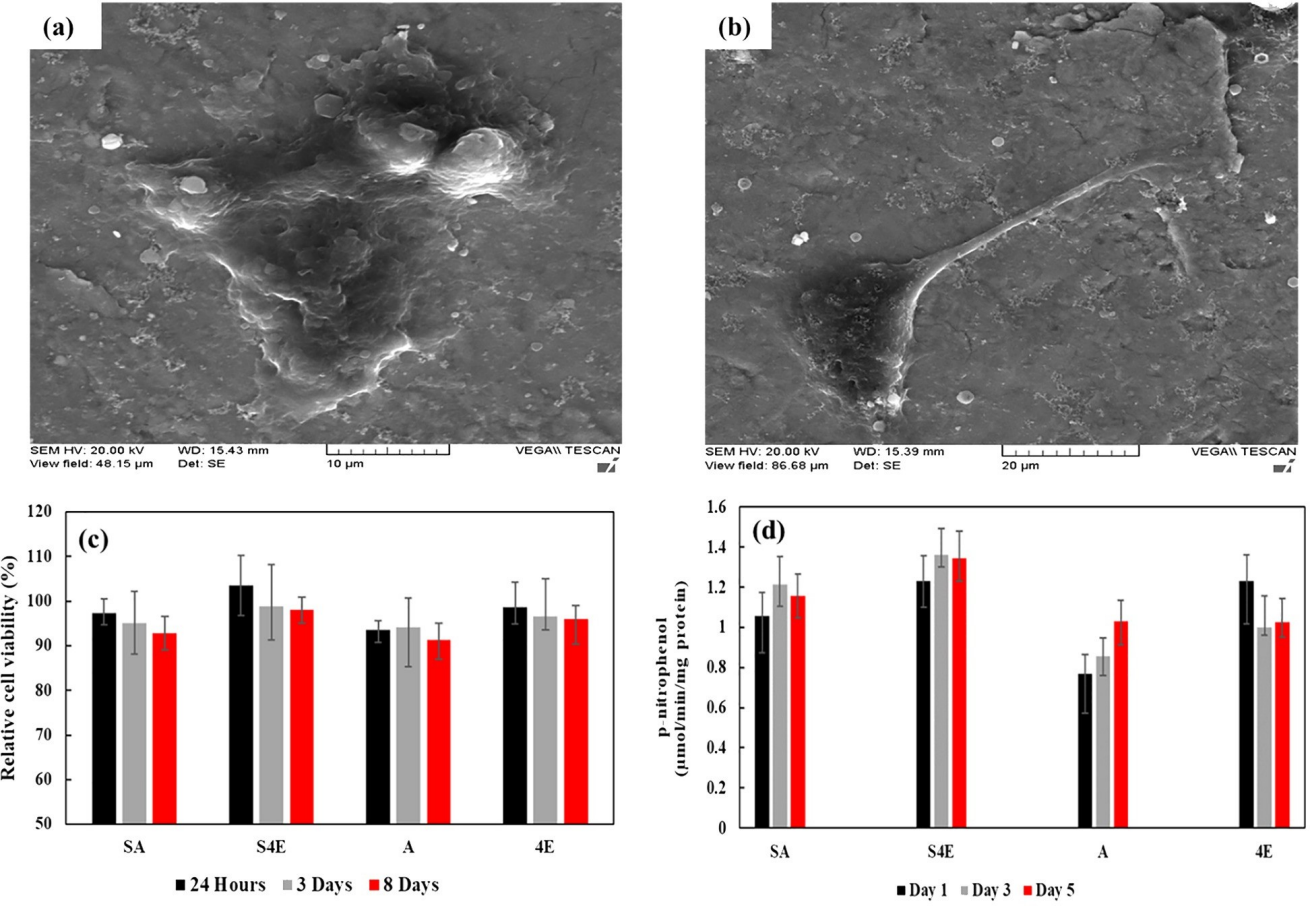
Osteoblast cell behavior of Ti samples; SEM images of G292 cells growing on Ti sample after 24 h of incubation: (a) A sample; (b) S4E sample; (c) relative cell viability of different titanium samples in 1, 3 and 8 culture days; (d) ALP activity of different titanium samples over 1, 3 and 5 days.

Fig 8 shows the osteoblast cell behavior results on Ti samples. Fig 8(A) and 8(B) shows the adhesion of the cells on the A and S4E samples. The cells on the S4E sample gradually adhered to surface and spread out on the substrate by the formation of filopods, indicating that SMATed + ECAPed sample can possibly show a better cell adhesion behavior compared to that of the annealed sample. It was reported [72] that formation of filopods can be a sign of improved cell adhesion. Fig 8(C) represents the results of cell viability test after culturing for 1, 3 and 8 days. The best results belong to S4E sample which shows higher values of cell viability when compared with sample A with the least value. Overall, the SMATed condition suggests a relatively higher cell viability than that of the non-SMATed condition which arises from different reasons such as improvement in the roughness, topography and wettability and even microstructural and crystallographic orientation of grains [73]. Although, in the non-SMATed condition, the ECAPed sample shows considerable better results than the as-received condition. It is assumed that grain refinement and UFG formation may cause such differences. In addition, the increased surface and grain boundary energy and possibly the existence of numerous nano-size conical groove-like structures generally found at triple point junctions of grain boundaries in the ECAP Ti sample can influence the biological behavior. This is also a responsible factor for better cellular behavior due to ECAP processing [74] and may be generalized for the SMAT case. Fig 8(D) represents the results of alkaline phosphatase (ALP) activity on different samples displaying the initial osteogenic differentiation tendency of the cells. It is clearly seen that SMAT process can positively elevate ALP activity after 1, 3 and 5 days in culture. Also, ECAPed sample shows a relatively higher ALP activity compared to that of annealed counterpart, similar trend was also previously reported on CP Ti [7]. In another study [75], as a result of grain refinement which influences cellular activity and biomineralization, nanostructured Ti showed better results for ALP activity.

## 4. Conclusions

ECAP was performed on CP Ti samples in 450°C up to four passes for attaining favorable mechanical bulk properties, then surface mechanical attrition treatment (SMAT) was applied in order to improve surface and mechanical properties, also biological experiments were done on different samples.

Three distinct zones with different microstructural properties was produced in SMATed samples. SMATed Annealed sample has a nanostructured layer with about 12.2 μm thickness. Beneath this nanostructured layer, there is a deformed layer with a high population of LAGBs. Four passes ECAPed sample also represents the formation of three layers with nanostructured zone in the topmost of sample and the deformed zone just beneath the first layer that it has a UFG core which is not affected by SMAT.

Approximately 89% grain refinement is achieved in S4E sample. The grain refinement process was attained through cDRX mechanism in ECAP sample since incomplete HAGB segments and necklace structure formation around initial grains was observed.

The nanoindentation hardness and reduced Young modulus (Er) results of SMATed samples also indicated the formation of graded zones and about 75% hardness improvement was attained in S4E sample in comparison with 4E sample.

SMAT processing induced 55% improvement in Rz roughness value in the as-received sample and about the 88% in ECAPed sample also wettability improved through SMAT processing and resulted in contact angle reduction which both are favorable for cell viability and adhesion. Also, SEM micrograph of SMATed samples indicated the formation of large number of pores and microcracks which are the preferred regions for cell proliferation and adhesion.

Viability, alkaline phosphate activity and cell attachment of G292 cell lines on different conditions of CP Ti confirms the beneficial effect of combined severe plastic deformation through the application of ECAP + SMAT process. Alkaline phosphate activity clearly showed that SMAT positively elevates the differentiation of cells. Also, the ECAPed sample shows a better ALP activity than the as-received counterpart.

The morphology of adhered cells on different Ti samples indicated that the cells did not spread out well on the surface of the as-received sample. By contrast, the cells on the SMATed sample shows the formation of filopods with a better cell adhesion behavior.

Application of SMAT process on ECAPed samples seems to be a very advantageous scheme to enhance biological, mechanical, surface and mechanical properties of CP Ti. The mentioned favorable characteristics due to bulk and surface optimization together, have a potential to produce modern and improved medical devices from metallic biomaterials.

## References

[1] Geetha M., Singh a. K., Asokamani R., and Gogia a. K., “Ti based biomaterials, the ultimate choice for orthopaedic implants–A review,” *Prog*. *Mater*. *Sci*., vol. 54, no. 3, pp. 397–425, 5 2009.

[2] Niinomi M., “Mechanical properties of biomedical titanium alloys,” *Mater*. *Sci*. *Eng*. *A*, vol. 243, no. 243, pp. 231–236, 1998.

[3] Sun Z. L., Wataha J. C., and Hanks C. T., “Effects of metal ions on osteoblast-like cell metabolism and differentiation,” *J*. *Biomed*. *Mater*. *Res*., vol. 34, pp. 29–37, 1997 8978650 10.1002/(sici)1097-4636(199701)34:1<29::aid-jbm5>3.0.co;2-p

[4] Gomes C. C. et al., “Assessment of the genetic risks of a metallic alloy used in medical implants,” *Genet*. *Mol*. *Biol*., vol. 34, no. 1, pp. 116–121, 2011 10.1590/S1415-47572010005000118 21637553 PMC3085356

[5] Valiev B. R. Z. et al., “Nanostructured Titanium for BiomedicalApplications,” *Adv*. *Eng*. *Mater*., vol. 10, no. 8, pp. 15–17, 2008.

[6] Estrin Y., Lapovok R., Medvedev A. E., Kasper C., Ivanova E., and Lowe T. C., “Mechanical performance and cell response of pure titanium with ultrafine-grained structure produced by severe plastic deformation,” in *Titanium in Medical and Dental Applications*, Elsevier Inc., 2018, pp. 419–454.

[7] Nie F. L. et al., “In vitro and in vivo studies on nanocrystalline Ti fabricated by equal channel angular pressing with microcrystalline CP Ti as control,” pp. 1694–1707, 2012 10.1002/jbm.a.34472 23184756

[8] Figueiredo R. B., Cetlin P. R., and Langdon T. G., “The processing of difficult-to-work alloys by ECAP with an emphasis on magnesium alloys,” *Acta Mater*., vol. 55, no. 14, pp. 4769–4779, 2007.

[9] Muley S. V., Vidvans A. N., Chaudhari G. P., and Udainiya S., “An assessment of ultra fine grained 316L stainless steel for implant applications,” *ACTA Biomater*., vol. 30, pp. 408–419, 2015 10.1016/j.actbio.2015.10.043 26518104

[10] Sunil B. R., Anil A., Kumar T. S. S., and Chakkingal U., “Role of biomineralization on the degradation of fine grained AZ31 magnesium alloy processed by groove pressing,” *Mater*. *Sci*. *Eng*. *C*, vol. 33, no. 3, pp. 1607–1615, 2013. 10.1016/j.msec.2012.12.095 23827614

[11] Zheng C. Y., Nie F. L., Zheng Y. F., Cheng Y., Wei S. C., and Valiev R. Z., “Enhanced in vitro biocompatibility of ultrafine-grained biomedical NiTi alloy with microporous surface,” *Appl*. *Surf*. *Sci*., vol. 257, no. 21, pp. 9086–9093, 2011.

[12] Valiev R. Z., Islamgaliev R. K., and V Alexandrov I., “Bulk nanostructured materials from severe plastic deformation,” *Prog*. *Mater*. *Sci*., vol. 45, pp. 103–189, 2000.

[13] Valiev R. Z. and Langdon T. G., “Principles of equal-channel angular pressing as a processing tool for grain refinement,” *Prog*. *Mater*. *Sci*., vol. 51, no. 7, pp. 881–981, 2006.

[14] Beygelzimer Y., Orlov D., and Varyukhin V., “A New Severe Plastic Deformation Method: Twist Extrusion,” *Ultrafine Grained Mater*. *II*, vol. The Minera, pp. 297–304, 2002.

[15] Ebrahimi M., Gholipour H., and Djavanroodi F., “A study on the capability of equal channel forward extrusion process,” *Mater*. *Sci*. *Eng*. *A*, vol. 650, pp. 1–7, 2015.

[16] Saito Y., Tsuji N., Utsunomiya H., Sakai T., and Hong R. G., “Ultra-fine grained bulk aluminum produced by accumulative roll-bonding (ARB) process,” *Scr*. *Mater*., vol. 39, no. 9, pp. 1221–1227, 10 1998.

[17] Ebrahimi M., Attarilar S., Djavanroodi F., Gode C., and Kim H. S., “Wear properties of brass samples subjected to constrained groove pressing process,” *Mater*. *Des*., vol. 63, pp. 531–537, 2014.

[18] Faraji G., Mosavi M., and Seop H., “Tubular channel angular pressing (TCAP) as a novel severe plastic deformation method for cylindrical tubes,” *Mater*. *Lett*., vol. 65, no. 19–20, pp. 3009–3012, 2011.

[19] Yin F. E. I., Hu S., Hua L. I. N., Wang X., and Suslov S., “Surface Nanocrystallization and Numerical Modeling of Low Carbon Steel by Means of Ultrasonic Shot Peening,” vol. 46, no. 3, pp. 1253–1261, 2015.

[20] Dai K. and Shaw L., “Comparison between shot peening and surface nanocrystallization and hardening processes,” *Mater*. *Sci*. *Eng*. *A*, vol. 463, pp. 46–53, 2007.

[21] Jin Z., Xingbin O. U., Donghua Y., and Zhifu S. U. N., “Surface Nanocrystallization of Magnesium Alloy AZ91D by High-Energy Shot Peening,” pp. 515–519, 2009.

[22] Lu K. and Lu J., “Nanostructured surface layer on metallic materials induced by surface mechanical attrition treatment,” *Mater*. *Sci*. *Eng*. *A*, vol. 377, pp. 38–45, 2004.

[23] Misra R. D. K., Nune C., Pesacreta T. C., Somani M. C., and Karjalainen L. P., “Understanding the impact of grain structure in austenitic stainless steel from a nanograined regime to a coarse-grained regime on osteoblast functions using a novel metal deformation–annealing sequence,” *Acta Biomater*., vol. 9, no. 4, pp. 6245–6258, 2013 10.1016/j.actbio.2012.12.003 23232208

[24] Bagherifard S. et al., “Effects of Nanofeatures Induced by Severe Shot Peening (SSP) on Mechanical, Corrosion and Cytocompatibility Properties of Magnesium Alloy AZ31,” *Acta Biomater*., no. 12, 2017. 10.1016/j.actbio.2017.11.032 29183850

[25] Valiev R. Z., Semenova I. P., Jakushina E., V Latysh V., and Rack H., “Nanostructured SPD Processed Titanium for Medical Implants,” *Mater*. *Sci*. *Forum*, vol. 586, pp. 49–54, 2008.

[26] Yao C., Slamovich E. B., Qazi J. I., Rack H. J., and Webster T. J., “Improved Bone Cell Adhesion on Ultrafine Grained Titanium and Ti‐6A1‐4V,” *Ceram*. *Nanomater*. *Nanotechnol*. *III*, vol. 159, pp. 239–245, 2005.

[27] Valiev R. Z., Sabirov I., Zemtsova E. G., V Parfenov E., Dluhos L., and Lowe T. C., “Nanostructured commercially pure titanium for development of miniaturized biomedical implants,” in *Titanium in Medical and Dental Applications*, Elsevier Inc., 2018, pp. 393–417.

[28] Kubacka D., Yamamoto A., Wieciński P., and Garbacz H., “Biological behavior of titanium processed by severe plastic deformation,” *Appl*. *Surf*. *Sci*., vol. 472, pp. 54–63, 2018.

[29] Bagheri S. and Guagliano M., “Review of shot peening processes to obtain nanocrystalline surfaces in metal alloys,” *Surf*. *Eng*., vol. 25, no. 1, pp. 3–14, 2009.

[30] Wennerberg A. and Albrektsson T., “Effects of titanium surface topography on bone integration: a systematic review,” *Clin*. *oral Implant*. *Res*. *J*., vol. 20, no. s4, pp. 172–184, 2009. 10.1111/j.1600-0501.2009.01775.x 19663964

[31] Wassmann T., Kreis S., Behr M., and Buergers R., “The influence of surface texture and wettability on initial bacterial adhesion on titanium and zirconium oxide dental implants,” *Int*. *J*. *Implant Dent*., vol. 32, no. 3, 2017. 10.1186/s40729-017-0093-3 PMC5511811 28714053

[32] Bahl S., Aleti B. T., Suwas S., and Chatterjee K., “Surface Nanostructuring of titanium imparts multifunctional properties for orthopedic and cardifcovascular applications,” *Mater*. *Des*., vol. 144, pp. 169–181, 2018.

[33] Yang D. K., Cizek P., Fabijanic D., Wang J. T., and Hodgson P. D., “Work hardening in ultrafine-grained titanium: Multilayering and grading,” *Acta Mater*., vol. 61, no. 8, pp. 2840–2852, 2013.

[34] Wenbo Liu P. C., Xiao Jin, Bo Zhang, Di Yun, “A Coupled EBSD / TEM Analysis of the Microstructure Evolution of a Gradient Nanostructured,” *Materials (Basel)*., vol. 12, no. 1, p. 140, 2019. 10.3390/ma12010140 PMC6337208 30609842

[35] Zhu K. Y., Vassel A., Brisset F., Lu K., and Lu J., “Nanostructure formation mechanism of a -titanium using SMAT,” *Acta Mater*., vol. 52, pp. 4101–4110, 2004.

[36] Taek K., Song K., Ho S., Lee Y., Mook K., and Beom W., “Surface hardening of aluminum alloy by shot peening treatment with Zn based ball,” *Mater*. *Sci*. *Eng*. *A*, vol. 543, pp. 44–49, 2012.

[37] Basu S., Wang Z., and Saldana C., “Deformation heterogeneity and texture in surface severe plastic deformation of copper,” *Proc*.*R*.*Soc*.*A*, vol. 472, no. 2187, pp. 1–15, 2016. 10.1098/rspa.2015.0486 PMC4841473 27118907

[38] Liu X., Yuan F., Zhu Y., and Wu X., “Extraordinary Bauschinger effect in gradient structured copper Scripta Materialia Extraordinary Bauschinger effect in gradient structured copper,” *Scr*. *Mater*., vol. 150, no. 5, pp. 57–60, 2018.

[39] Wu X., Jiang P., and Chen L., “Synergetic Strengthening by Gradient Structure Synergetic Strengthening by Gradient Structure,” *Mater*. *Res*. *Lett*., vol. 2, no. 4, pp. 185–191, 2014.

[40] Stolyarov V. V., Theodore Zhu Y., Alexandrov I. V., Lowe T. C., and Valiev R. Z., “Influence of ECAP routes on the microstructure and properties of pure Ti,” *Mater*. *Sci*. *Eng*. *A*, vol. 299, no. 1–2, pp. 59–67, 2001.

[41] Heydari A., Miresmaeili R., Bagherifard S., Guagliano M., and Aliofkhazraei M., “Incorporating the principles of shot peening for a better understanding of surface mechanical attrition treatment (SMAT) by simulations and experiments,” *Mater*. *Des*., vol. 116, pp. 365–373, 2017.

[42] Unal O., Cahit Karaoglanli A., Varol R., and Kobayashi A., “Microstructure evolution and mechanical behavior of severe shot peened commercially pure titanium,” *Vacuum*, vol. 110, pp. 202–206, 2014.

[43] Shuman D. J., Costa A. L. M., and Andrade M. S., “Calculating the elastic modulus from nanoindentation and microindentation reload curves,” *Mater*. *Charact*., vol. 58, no. 4, pp. 380–389, 2007.

[44] Oliver W. C. and Pharr G. M., “An improved technique for determining hardness and elastic modulus using load and displacement sensing indentation experiments,” *J*. *Mater*. *Res*., vol. 7, no. 6, pp. 1564–1583, 1992.

[45] Sakai T., Belyakov A., Kaibyshev R., Miura H., and Jonas J. J., “Dynamic and post-dynamic recrystallization under hot, cold and severe plastic deformation conditions,” *Prog*. *Mater*. *Sci*., vol. 60, pp. 130–207, 2014.

[46] Bieda M. et al., “Microstructure of titanium on complex deformation paths: Comparison of ECAP, KOBO and HE techniques,” *Mater*. *Charact*. *J*., vol. 141, no. 1, pp. 19–31, 2018.

[47] HUMPHREYS F. J. and HATHERLY M., *Recrystallization and Related Annealing Phenomena*, 2th ed Elsevier Ltd, 2004.

[48] Chen Y. J., Li Y. J., Walmsley J. C., Dumoulin S., Skaret P. C., and Roven H. J., “Microstructure evolution of commercial pure titanium during equal channel angular pressing,” *Mater*. *Sci*. *Eng*. *A*, vol. 527, no. 3, pp. 789–796, 2010.

[49] Al-Samman T. and Gottstein G., “Dynamic recrystallization during high temperature deformation of magnesium,” *Mater*. *Sci*. *Eng*. *A*, vol. 490, no. 1–2, pp. 411–420, 8 2008.

[50] Chen Y. J., Li Y. J., Walmsley J. C., Dumoulin S., and Roven H. J., “Deformation structures of pure titanium during shear deformation,” *Metall*. *Mater*. *Trans*. *A Phys*. *Metall*. *Mater*. *Sci*., vol. 41, no. 4, pp. 787–794, 2010.

[51] Yan H., Bi H., Li X., and Xu Z., “Microstructure, texture and grain boundaries character distribution evolution of ferritic stainless steel during rolling process,” *J*. *Mater*. *Process*. *Technol*., vol. 209, no. 5, pp. 2627–2631, 2009.

[52] Estrin Y. and Vinogradov A., “Extreme grain refinement by severe plastic deformation: A wealth of challenging science,” *Acta Mater*., vol. 61, no. 3, pp. 782–817, 2013.

[53] Hashmi S., *COMPREHENSIVE MATERIALS PROCESSING*. 2014.

[54] Moskalenko V. A., Smirnov A. R., Plotnikova Y. M., Braude I. S., and Smolianets R. V., “Fundamentals of titanium nanocrystalline structure creation by cryomechanical grain fragmentation,” *Mater*. *Sci*. *Eng*. *A*, vol. 700, no. 6, pp. 707–713, 2017.

[55] Qarni M. J., Sivaswamy G., Rosochowski A., and Boczkal S., “On the evolution of microstructure and texture in commercial purity titanium during multiple passes of incremental equal channel angular pressing (I-ECAP),” *Mater*. *Sci*. *Eng*. *A*, vol. 699, no. January, pp. 31–47, 2017.

[56] Sorensen C., Basinger J. A., Nowell M. M., and Fullwood D. T., “Five-parameter grain boundary inclination recovery with ebsd and interaction volume models,” *Metall*. *Mater*. *Trans*. *A Phys*. *Metall*. *Mater*. *Sci*., vol. 45, no. 9, pp. 4165–4172, 2014.

[57] Abbasi M., Kim D.-I., Guim H., Hosseini M., and Abbasi H. D.-M. M., “Application of Transmitted Kikuchi Diffraction in Studying Nano-oxide and Ultrafine Metallic Grains,” *ACS Nano*, vol. 9, no. 11, pp. 10991–11002, 2015 10.1021/acsnano.5b04296 26482120

[58] Beausir B. and Fundenberger J.-J., “Analysis Tools for Electron and X-ray diffraction, ATEX—software, Université de Lorraine,” *www.atex-software.eu*, 2017.

[59] Wang Y., “Fundamentals of Recrystallization in Titanium Alloys,” 2014.

[60] Gadelmawla E. S., Koura M. M., Maksoud T. M. A., Elewa I. M., and Soliman H. H., “Roughness parameters,” *J*. *Mater*. *Process*. *Technol*., vol. 123, pp. 133–145, 2002.

[61] Bagherifard S., Ghelichi R., Khademhosseini A., and Guagliano M., “Cell Response to Nanocrystallized Metallic Substrates Obtained through Severe Plastic Deformation,” *Appl*. *Mater*. *Interfaces*, vol. 6, pp. 7963–7985, 2014. 10.1021/am501119k 24755013

[62] Ponsonnet L. et al., “Relationship between surface properties (roughness, wettability) of titanium and titanium alloys and cell behaviour,” *Mater*. *Sci*. *Eng*. *C*, vol. 23, no. 4, pp. 551–560, 2003.

[63] Bornstein M. M., Jones A. A., Wilson T. G., and Cochran D. L., “Bone apposition around two different sandblasted and acid-etched titanium implant surfaces: a histomorphometric study in canine mandibles,” *Clin*. *Oral Impl*. *Res*, vol. 19, pp. 233–241, 2008. 10.1111/j.1600-0501.2007.01473.x 18177427

[64] Dds Y. S. and Tanimoto Y., “A review of improved fixation methods for dental implants. Part I: Surface optimization for rapid osseointegration,” *J*. *Prosthodont*. *Res*., vol. 59, no. 1, pp. 20–33, 2014 10.1016/j.jpor.2014.11.007 25530606

[65] Zheng C. Y., Nie F. L., Zheng Y. F., Cheng Y., Wei S. C., and Valiev R. Z., “Enhanced in vitro biocompatibility of ultrafine-grained titanium with hierarchical porous surface,” *Appl*. *Surf*. *Sci*., vol. 257, no. 13, pp. 5634–5640, 4 2011.

[66] Huang L., Lu J., and Troyon M., “Nanomechanical properties of nanostructured titanium prepared by SMAT,” vol. l, pp. 208–213, 2006.

[67] Meyers M. A., Mishra A., and Benson D. J., “Mechanical properties of nanocrystalline materials,” *Prog*. *Mater*. *Sci*., vol. 51, no. 4, pp. 427–556, 2006.

[68] Würschum B. R., Herth S., and Brossmann U., “Diffusion in Nanocrystalline Metals and Alloys-A Status Report,” *Adv*. *Eng*. *Mater*., vol. 5, pp. 365–372, 2003.

[69] Zhu Y. T., Lowe T. C., and Langdon T. G., “Performance and applications of nanostructured materials produced by severe plastic deformation,” *Scr*. *Mater*. *51*, vol. 51, pp. 825–830, 2004.

[70] Pougis A., Toth L. S., Fundenberger J. J., and Borbely A., “Extension of the Derby relation to metals severely deformed to their steady-state ultrafine-grain size,” *Scr*. *Mater*., vol. 72–73, pp. 59–62, 2014.

[71] Zhu Y. T. and Liao X., “Retaining ductility,” *Nanostructured Met*., vol. 3, pp. 351–352, 2004. 10.1038/nmat1141 15173850

[72] Hahn B. D. et al., “Mechanical and in vitro biological performances of hydroxyapatite-carbon nanotube composite coatings deposited on Ti by aerosol deposition,” *Acta Biomater*., vol. 5, no. 8, pp. 3205–3214, 2009 10.1016/j.actbio.2009.05.005 19446047

[73] Faghihi S. et al., “The significance of crystallographic texture of titanium alloy substrates on pre-osteoblast responses,” *Biomaterials*, vol. 27, pp. 3532–3539, 2006 10.1016/j.biomaterials.2006.02.027 16545866

[74] Kim T. N., Balakrishnan A., Lee B. C., Kim W. S., and Smetana K., “In vitro biocompatibility of equal channel angular processed (ECAP) titanium,” *Biomed*. *Mater*., vol. 2, no. July 2016, pp. S117–S120, 2007 10.1088/1748-6041/2/3/S06 18458454

[75] Ferreira M. R. W. et al., “Oxidative Nanopatterning of Titanium Surface Influences mRNA and MicroRNA Expression in Human Alveolar Bone Osteoblastic Cells,” *Int*. *J*. *Biomater*., vol. 2016, no. 4, pp. 1–15, 2016. 10.1155/2016/9169371 PMC4856946 27200092

